# Membrane-Based Clarification and Fractionation of Red Wine Lees Aqueous Extracts

**DOI:** 10.3390/polym11071089

**Published:** 2019-06-26

**Authors:** Alfredo Cassano, Antonella Bentivenga, Carmela Conidi, Francesco Galiano, Omar Saoncella, Alberto Figoli

**Affiliations:** Institute on Membrane Technology, ITM-CNR, c/o University of Calabria, via P. Bucci, 17/C, I-87036 Rende (Cosenza), Italy

**Keywords:** winery effluents, bioactive compounds, membrane-based operations, biorefinery

## Abstract

Polyvinylidenefluoride (PVDF) hollow fiber membranes prepared in laboratory through the inversion phase method were characterized and used to clarify an aqueous extract from red wine lees. Steady-state permeate fluxes of 53 kg/m^2^h were obtained in the treatment of the aqueous extract in selected operating conditions. Suspended solids were completely retained by the hollow fiber membranes while bioactive compounds, including polyphenols, anthocyanins, and resveratrol were recovered in the permeate stream. The clarified stream was then fractionated by nanofiltration (NF). Three different commercial membranes, in flat-sheet configuration (NP010 and NP030 from Microdyn-Nadir, MPF36 from Koch Membrane Systems), were selected and tested for their productivity and selectivity towards sugars and bioactive compounds, including phenolic compounds, anthocyanins, and resveratrol. All selected membranes showed high retention towards anthocyanins (higher than 93%). Therefore, they were considered suitable to concentrate anthocyanins from clarified wine lees extracts at low temperature. On the other hand, NF permeate streams resulted enriched in phenolic compounds and resveratrol. Among the selected membranes, the MPF36 exhibited the lowest retention towards resveratrol (10%) and polyphenols (26.3%) and the best separation factor between these compounds and anthocyanins.

## 1. Introduction

The winemaking industry generates different residues characterized by high contents of biodegradable compounds and suspended solids [1]. In particular, the main solid by-products and wastes produced during winemaking are grape stalks, grape marc, and wine lees.

Wine lees are deposits of dead yeasts, particulates and other precipitates formed at the bottom of wine containers after fermentation, during storage or after wine filtration. They represent approx. 2–6% of the total volume of wine produced [2]. These wastes have a potential use as a supplement in animal feed. However, the main drawback at this purpose is the low nutrient value of yeasts recovered by centrifugation after column distillation. [3].

Conventional treatments of winery wastes are increasingly expensive, requiring significant amounts of effort, resources, and energy for safe discharge into the environment. On the other hand, wine lees contain phenolic compounds including low molecular weight acids, tannins, flavonols, anthocyanins, and resveratrol, well recognized for their potential beneficial effects on human health due to their antioxidant, antimicrobial, antiviral and anti-inflammatory activity [4]. Therefore, their integration into functional foods, nutraceuticals, and cosmetics has attracted both researchers and industries to identify new technological approaches for their recovery in order to provide additional sources of income from their industrial exploitation and, at the same time, viable solutions to reduce the waste disposal [5].

Conventionally, the recovery of phenolic compounds from agro-food wastes and plant materials is based on the use of maceration assisted by liquid solvents [6], thus requiring long extraction times. Microwave, pressurized liquid extraction, and ultra-sonic extraction methods have been also proposed [7,8,9]. However, these methodologies are characterized by high energy costs and lead to the production of excessive solvent waste, which can be more hazardous to dispose of than the actual agricultural waste itself.

In this context, the development of “green” extraction and separation procedures is of great importance into industrial processes to reduce or eliminate the use and generation of hazardous substances. Innovative green analytical metrics, such as analytical eco-scale [10] and green analytical procedure Index [11], have been also proposed as comprehensive approaches to evaluating the greenness of analytical methodologies.

The use of water as cheap, non-hazardous polar extraction solvent has attracted much interest in recent years. It has been shown to efficiently extract a wide variety of antioxidant compounds from plant materials [12]. Similarly, membrane processes play a key role in green chemistry due to their low operating and maintenance costs, no use of chemical additives, mild operating conditions of temperature and pressure, easy control and scale-up, and high selective separations [13]. Their intrinsic characteristics of selectivity, modularity, efficiency, reduced energy consumption, and waste production, decreased equipment size/productivity ratio, increased safety, remote control and automation well fit the requirements of green and intensified processes [14].

Giacobbo et al. [15] investigated the use of microfiltration (MF) for the recovery of polyphenols from wine lees generated during the second racking, with or without using previous water addition of the effluent. MF, ultrafiltration (UF) and nanofiltration (NF) in sequential design were also investigated in order to fractionate polyphenols and polysaccharides from wine lees [16]. According to the proposed flow-sheet the wine lees extract was pretreated by MF in order to remove suspended colloidal particles. UF and NF membranes allowed to produce concentrated fractions of polysaccharides and polyphenols from the MF permeate and the UF permeate, respectively.

Kontogiannopoulos et al. [17] evaluated the performance of various UF and NF membranes for their efficiency in separating tartaric acid and phenolic compounds from a liquid stream obtained after the centrifugation of an acidified solution of dry lees containing a cation exchange resin (3.5 g resin and 10 mL of acidified water at pH 3.0 per g of dry lees). This approach was mainly focused on the dissolution and recovery of tartaric acid from wine lees.

Galanakis et al. [18] evaluated the performance of UF membranes for the separation and fractionation of phenolic compounds from hydro-ethanolic extracts of wine lees, collected after the first decanting stage. Among the investigated membranes, 1 kDa fluoropolymer membranes separated successfully hydroxycinnamic acid derivatives from anthocyanins and flavonols. On the other hand, 100 kDa polysulphone membranes were not capable of fractionating phenolic classes but allowed their separation from pectin and hydrolyzed derivatives.

Sustainable extractive technologies followed by membrane separation methods have been recently investigated to obtain commercial extracts of interest in the functional food industry, pharmaceutical and cosmetic fields from olives and artichoke by-products [19,20].

In the light of the aforementioned investigations, a combination of aqueous extraction and membrane operations was investigated on laboratory scale in order to develop a sustainable and environment-friendly approach for the extraction and purification of bioactive compounds from wine lees, in agreement with the principles of green chemistry. Specifically, the crude extract obtained from wine lees through an aqueous extraction at 45 °C was clarified by using poly(vinylidenefluoride) (PVDF) hollow fibers (HFs), prepared according to the phase inversion process. The clarified extract was then processed with three different commercial membranes in flat-sheet configuration and different molecular weight cut-off (MWCO) in order to evaluate and compare their potential to obtain high added-value extracts of interest in the formulation of natural antioxidant-based products. The performance of selected membranes was evaluated in terms of productivity and selectivity towards total phenols, anthocyanins, sugars, resveratrol and total antioxidant activity.

## 2. Materials and Methods

### 2.1. Reagents

Polyvinylidenefluoride (PVDF) was Solef^®^6010 supplied by Solvay Specialty Polymers (Milan, Italy). It is a white powder with a density of 1.78 gr/m^3^ and a melting temperature point between 165 and 177 °C. PVDF was chosen because it is an inert material suitable for use in contact with food products. Dimethylformammide (DMF), supplied by Sigma Aldrich (Milan, Italy), was used as a solvent for the solution. Polyvinilpyrrolidone (PVP, Luviskol K17, MW = 12 kg/mol) was supplied by BASF (Ludwigshafen, Germany). Glycerol was supplied by Carlo Erba Reagents (Milan, Italy).

### 2.2. Preparation of PVDF HF Membranes

PVDF HF membranes were prepared by the dry/wet spinning technique described by Figoli et al. [21]. The polymeric dope was prepared by dissolving PVDF Solef^®^6010 25 wt.% in DMF. PVP Luviskol K17 was used as pore-forming additive, in the concentration of 35 wt.%. It was desiccated at 50 °C under vacuum overnight before use. The detailed conditions of HF spinning are resumed in Table 1.

The produced HFs were washed in distilled water at 50 °C for at least 24 h in order to remove residual solvent and soluble additive (PVP). Then, they were soaked in a glycerol/water solution (40 wt.%) for other 24 h in order to prevent pore collapse and finally dried at room temperature for 48 h.

### 2.3. HFs Characterization

PVDF HFs morphology was evaluated by scanning electron microscopy (SEM) (Quanta FENG 200, FEI Co., Hillsboro, OR, USA). Cross-sections were freeze-fractured in liquid nitrogen.

The mechanical properties of the HFs (Young’s modulus (*ε_m_*), tensile stress at break (*R_m_*) and elongation at break (%) were measured using a ZWICK/ROELL Z 2.5 test unit, as described elsewhere [21]. The reported values were calculated as an average of five measurements. The porosity was calculated by the gravimetric method [22]: Three dry HFs samples were weighed and subsequently soaked in kerosene for 24 h. The weight was, then, measured again.

The average void fraction was calculated according to the following equation:

(1) $$\varepsilon_{m}\left(\%\right)=\frac{\frac{w_{1}-w_{2}}{D_{k}}}{\frac{w_{1}-w_{2}}{D_{k}}+\frac{w_{2}}{D_{pol}}}\cdot 100$$

where *w*_1_ is the weight of the wet membrane, *w*_2_ is the weight of the dry membrane, *D_k_* is the kerosene density (0.82 g/cm^3^), and *D_pol_* is the PVDF density (1.78 g/cm^3^).

A capillary flow porometer (CFP 1500 AEXL, PMI porous materials Inc., Ithaca, NY, USA) was used for determining the pore size of the HFs, as described elsewhere [21]. Tests were carried using the wet up/dry up method and using Porewick (16 dyne/cm) as wetting liquid.

Pure water permeability (PWP) was determined by using a bench plant (DSS Lab Unit M 10) supplied by Danish Separation System (Nakskov, Denmark) equipped with a membrane module prepared by embedding three HF membranes inside a 20 cm long glass tube (effective membrane length 175 mm, effective membrane area 22.76 cm^2^) with epoxy resin (Stycast, Emerson and Cuming, Belgium).

Water permeability set-up was composed of a feed tank, a cross-flow pump (ECO type GA4-KDT-TTU), two manometers located at the entrance (P_in_) and at the exit (P_out_) of the membrane module, a pressure control valve and a multitube heat exchanger fed with tap water. The water permeability was calculated from the slope of the straight lines fitted in the plot reporting the relationship between transmembrane water flux (J) and transmembrane pressure (TMP).

*J* (expressed in kg/m^2^h) was calculated by using the following equation:

(2) $$J=\frac{Q}{t\cdot A_{s}}$$

where Q is the volume of collected permeate (L), A_s_ is the active membrane surface (m^2^), t is the operating time (h).

### 2.4. Aqueous Extract of Red Wine Lees

Red wine lees (Nero d’Avola variety) samples were collected during the 2015–2016 season from the bottom of a stainless steel wine stabilization tank. The collected wine lees were immediately transferred to the laboratory and stored in a freezer (−17 °C). After defrosting, about 800 g of wine lees were diluted with distilled water (25% *w*/*w*) and thermostated in a water bath at 45 °C for 1h . The aqueous extract was filtered on nylon cloth, and the procedure was then repeated until to obtain a final volume of 6.85 L of aqueous extract. It was used as feed solution in the clarification step.

### 2.5. Clarification of Aqueous Extract

The aqueous extract was clarified by using the same equipment used for the measurement of the water permeability of HF membranes. Preliminary experiments were performed according to the total recycle configuration (recycling both permeate and retentate streams in the feed reservoir) in order to evaluate the effect of TMP on the permeate flux.

MF experiments were performed at an axial feed flowrate (Q_f_) of 580 mL/min and a temperature of 24 ± 2 °C. TMP was investigated in the range 0.3–1.2 bar. The duration of each experiment was 30 min.

The aqueous extract was clarified according to a batch concentration configuration (collecting separately the permeate stream and recycling the retentate stream in the feed reservoir) at a TMP of 0.8 bar, a Q_f_ of 580 mL/min and a temperature of 24 ± 2 °C up to a weight reduction factor (WRF) of 4.5.

The permeate stream was used as a feed solution in the fractionation step.

After the treatment of the aqueous extract, PVDF membranes were cleaned with distilled water (at 40 °C for 30 min) in order to remove the polarized layer on the membrane surface. Then the fibers were cleaned with a solution of sodium hypochlorite (1%, 60 min, 40 °C) followed by a cleaning step with an enzymatic solution (Ultrasil P3, 1%, 60 min, 40 °C).

The fouling index (FI) of PVDF membranes, expressed as percentage drop in water permeability, was estimated by measuring the water permeability before and after the treatment of wine lees extract, according to the following equation:

(3) $$FI=\left(1-\frac{PWP_{1}}{PWP_{0}}\right)\times 100$$

where PWP_0_ and PWP_1_ are the water permeabilities measured before and after the treatment of the aqueous extract.

The cleaning efficiency (CE) was evaluated by using the water flux recovery method, according to the following equation:

(4) $$CE=\left(\frac{PWP_{4}}{PWP_{0}}\right)\cdot 100$$

where PWP_4_ is the water permeability measured after the enzymatic cleaning.

### 2.6. Fractionation of Clarified Extract

The clarified extract was fractionated by using three different flat-sheet NF membranes with a molecular weight cut-off in the range 400–1000 Da. Membrane codes and technical specifications are reported in Table 2.

Filtration experiments were performed in dead-end filtration mode by using an experimental set-up comprising a high-pressure stirred cell filtration (SterlitechTM HP 4750, Kent, WA, USA) with an effective membrane area of 13.85 cm^2^ and a processing capacity of 300 mL. It is capable of operating up to a maximum pressure of 68.9 bar. The stirred cell assembly consists of three primary components: A cylindrical body with removable top and bottom, a Teflon-coated stir bar assembly to provide mixing and a porous stainless steel membrane support disk. A nitrogen cylinder, equipped with a two-stage pressure regulator, was connected to the top of the stirred cell to supply the desired pressure for filtration experiments. The prevailing transmembrane pressure (TMP) was monitored by a manometer connected at the cell inlet. Stirring inside the cell was accomplished by using a magnetic stirrer.

All experiments were performed by using 200 mL of a clarified extract with constant fluid agitation (350 rpm), at an operating pressure of 20 bar and at a temperature of 25 ± 2 °C, until to reach a WRF of 2.0.

**Figure 1:**
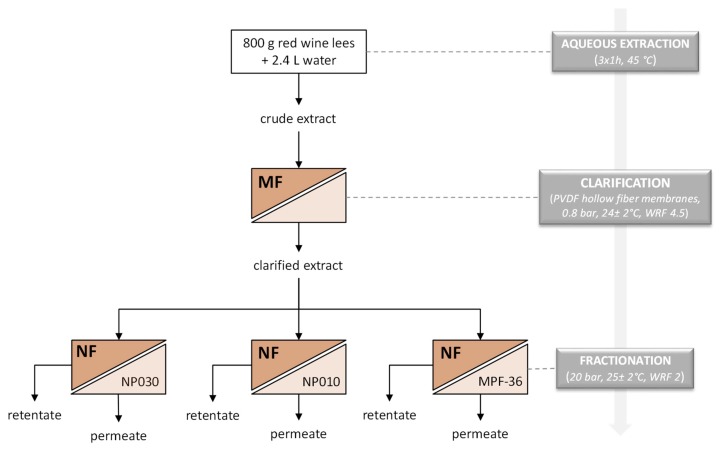
Schematic representation of the experimental design.

A schematic representation of the procedure used in this study is illustrated in Figure 1.

### 2.7. Analytical Measurements

Feed (F), permeate (P) and retentate (R) samples coming from filtration experiments were collected and analyzed in terms of suspended solids, total soluble solids, total polyphenols, total carbohydrates, total anthocyanins, and resveratrol.

Suspended solids were determined by centrifuging 10 mL of a pre-weight sample at 2000 rpm for 20 min. The weight of settled solids was determined after removing the supernatant.

Total soluble solids (TSS) measurements were carried out using a hand refractometer (Atago Co., Ltd., Tokyo, Japan) with a scale range of 0–32 °Brix.

The total polyphenols content was estimated by using the Folin–Ciocalteau method [25]. In the experiment, 0.2 mL of each sample were mixed with 1 mL of Folin–Ciocalteu reagent (diluted 10 times with bidistilled water). After 5 min, 2.5 mL of sodium carbonate solution (7.5%) were added and the mixture was allowed to stand for 30 min. The absorbance of the resulting solution was measured at 765 nm by using a UV-visible spectrophotometer (Shimadzu UV-160A, Kyoto, Japan). Gallic acid was used as a calibration standard and results were expressed as gallic acid equivalent (mgGAE/L).

Total carbohydrates were measured by using the phenol-sulfuric acid method [26]. A sample aliquot (0.2 mL) of a carbohydrate solution is mixed with 1 mL of 5% aqueous solution of phenol in a test tube. Then, 5 mL of concentrated sulfuric acid is added rapidly to the mixture. After allowing the test tubes to stand for 10 min, they are vortexes for 30 s and placed for 30 min in a water bath at room temperature for color development. The absorbance was measured at 420 min by using an UV-visible spectrophotometer (Shimadzu UV-160 A, Japan). Glucose solutions with concentrations ranging from 0.02 e 0.1 g/L were used for calibration. A dose response linear regression was generated by using the glucose standard absorbance, and results were expressed as g/L of glucose.

The total antioxidant activity (TAA) was determined by an improved version of the 2,2-azino-bis-(3-ethylbenzothiazoline-6-sulfonic acid) diammonium salt (ABTS) assay [27] in which the ABTS radical cation is generated by reaction with potassium persulphate (K_2_S_2_O_8_) before the addition of the antioxidant [28]. The decolorisation of the blue/green radical cation was measured as the percentage inhibition of absorbance at 734 nm. Results were expressed in terms of mM Trolox equivalents. Each determination was performed in triplicate. Results were expressed as mean ± SD of three samples.

The determination of total anthocyanins was carried out by spectrophotometric measurements [29,30]. Spectrophotometric analyses were performed under the following conditions: 5 mL of sample were added to 40 mL of an EtOH/HCl mixture previously prepared by mixing 79.7 mL of anhydrous ethyl alcohol with 20.3 mL of HCl (37%). The absorbance was measured at 535 nm by using a UV-Vis 160A Shimadzu spectrophotometer. The calibration curve was obtained by measuring the absorbance of standard solutions of pure cyanidin-3 glucoside. Concentrations were estimated using an extinction coefficient of 1018.3. Measurements were made in triplicate.

Resveratrol was assessed by high-performance liquid chromatography (HPLC) using a D-7000 HPLC system manager (Hitachi) equipped with a vacuum degasser, a quaternary pump, an autosampler, and a UV-Vis diode array detector. Chromatographic separation was performed by using a Luna C18(2) column (250m m × 4.60 mm, 5 μm, Phenomenex, Torrance, CA, USA) according to the following conditions: V = 1 mL/min, T = 25 °C, pressure = 90 bar, λ = 320 nm. Samples were eluted in isocratic mode by using a mixture of water/acetonitrile/acetic acid (70/29.9/0.1 *v*/*v/v*) [31].

The peak areas in the chromatograms were plotted against calibration curves obtained from resveratrol standard solutions (external standard method) in a concentration range of 50–300 ppm.

The rejection of selected membranes towards specific compounds was calculated according to the following equations, respectively:

(5) $$R=\left(1-\frac{c_{p}}{c_{f}}\right)\cdot 100\;$$

where c_p_ and c_f_ are the permeate and feed solute concentrations, respectively.

## 3. Results and discussion

### 3.1. HFs Morphology

**Figure 2:**
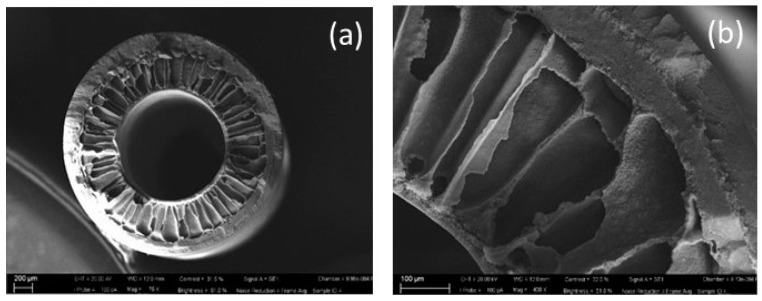
Cross section (**a**) and enlarged cross section (**b**) of PVDF HF membranes.

The cross-section of HF membranes showed a typical symmetric structure, mainly made up of parallel finger-like macrovoids (Figure 2a,b). This structure is a result of the balance between thermodynamic and kinetic factors during coagulation including the viscosity of the dope, viscosity, dimensions and surface tension of solvent and non-solvent molecules and mutual affinity (Hildebrand’s parameters) between polymer, solvent, non-solvent, and temperature [32]. These variables can be at the bases of the promotion or hindrance of the liquid/liquid demixing occurring during the formation of the membrane affecting its morphology (in particular influencing the formation of macrovoids).

The high percentage of PVP in the dope (35 wt.%) strongly increases its instability promoting faster coagulation and enhancing macrovoids growth (thermodynamic effect).

In addition, the high percentage of both PVP and PVDF in the dope solution leads to very high viscosity at room temperature, which should delay the demixing process as a consequence of the kinetic hindrance. However, the HFs were spun at the temperature of 120 °C where the dope solution appeared visually fluid. Therefore, kinetic hindrance of macrovoids formation was not observed.

**Figure 3:**
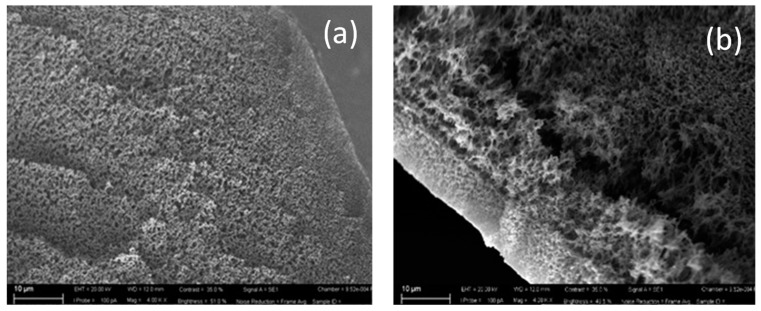
Cross section of inner (**a**) and outer (**b**) surface of PVDF HF membranes.

As shown in Figure 3, the HFs presented a sponge-like structure at both internal (Figure 3a) and external (Figure 3b) surface comprising a central structure made up of finger-like macrovoids. Similar architecture has been already observed for PVDF membranes prepared with PVP and NMP, as solvent, by Figoli et al. [21]. The inner coagulant plays an important role in determining HF inner morphology. In this study, the presence of solvent (DMF) in the bore fluid slowed down the demixing rate replacing the macrovoids with a sponge-like structure [22].

### 3.2. Mechanical Properties, Porosity, Pore Size and Pure Water Permeability of HF Membranes

The main properties of the prepared PVDF HFs are resumed in Table 3. HFs were characterized by a thickness of 0.49 ± 0.9 mm, an outer diameter of 2.29 ± 0.54 mm and an inner diameter of 1.30 ± 0.65 mm. HFs were also characterized by reduced mechanical strength, reflected by both low Young’s modulus and tensile stress at break. At the same time, the produced HFs showed very high porosity (81.0 ± 4.5 %). These characteristics can be explained taking into account their finger-like morphology. As reported in previous work [33] a typical trade-off between void fraction and mechanical properties was observed. The macrovoids represent, in fact, weak points into the membrane matrix leading to a decrease of its mechanical resistance [34,35].

The produced fibers showed an average pore diameter of 0.13 μm which is typical of MF membranes. As reported in the literature, larger pore size can be obtained for fibers spun using water/solvent mixture as an inner coagulant and with slow solvent/non-solvent exchange [36].

The measured water permeability of prepared membranes was of about 396 kg/m^2^hbar, in agreement with typical values of MF membranes.

### 3.3. Clarification of Wine Lees Aqueous Extract

**Figure 4:**
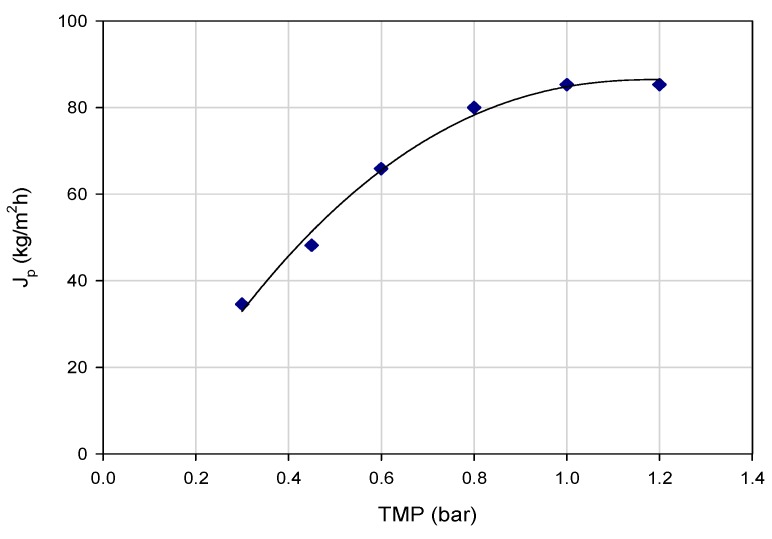
Clarification of wine lees extract with PVDF HF membranes. Permeate flux as a function of TMP (Operating conditions: Q_f_, 580 mL/min; T, 24 ± 2 °C).

The effect of TMP on the permeate flux was evaluated in experiments performed according to the total recycle configuration at an axial feed flowrate (Q_f_) of 580 mL/min and a temperature of 24 ± 2 °C. Figure 4 shows the permeate flux values, at steady-state, as a function of the applied TMP: At lower pressures, the permeate flux increases linearly with the applied pressure (range 0.3–0.6 bar). The rate of increase in flux decreases at higher pressures and a limiting flux due to the formation of a gelatinous-type layer on the membrane surface was reached at a TMP of 1.0 bar.

**Figure 5:**
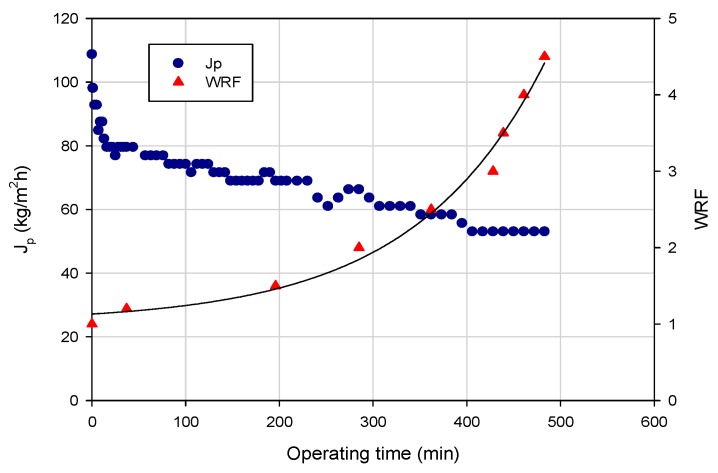
Clarification of wine lees extract with PVDF hollow fiber membranes. Time course of permeate flux and WRF (Operating conditions: TMP, 0.8 bar; Q_f_, 580 mL/min; T, 24 ± 2 °C).

Figure 5 shows the time evolution of permeate flux and WRF in the clarification of the wine lees extract with the HFs according to the batch concentration configuration. The aqueous extract was processed at a TMP of 0.8 bar, a Qf of 580 mL/min and a temperature of 24 ± 2 °C up to a weight reduction factor (WRF) of 4.5.

The initial permeate flux, of about 108 kg/m^2^h, decreased gradually with the operating times by increasing the volume reduction factor up to reach a steady-state value of 53 kg/m^2^h. This behavior can be attributed to different phenomena which include concentration polarization, membrane fouling and increased concentration of solutes in the retentate stream. Indeed, as the feed concentration increases, the concentration polarization becomes more severe. More solutes are convected towards the membrane surface, resulting in a thicker cake layer.

Steady-state permeate fluxes resulted similar to those reported by Simone et al. [33] in the clarification of orange press liquors with PVDF hollow fiber membranes with a pore diameter of 0.22 μm.

**Figure 6:**
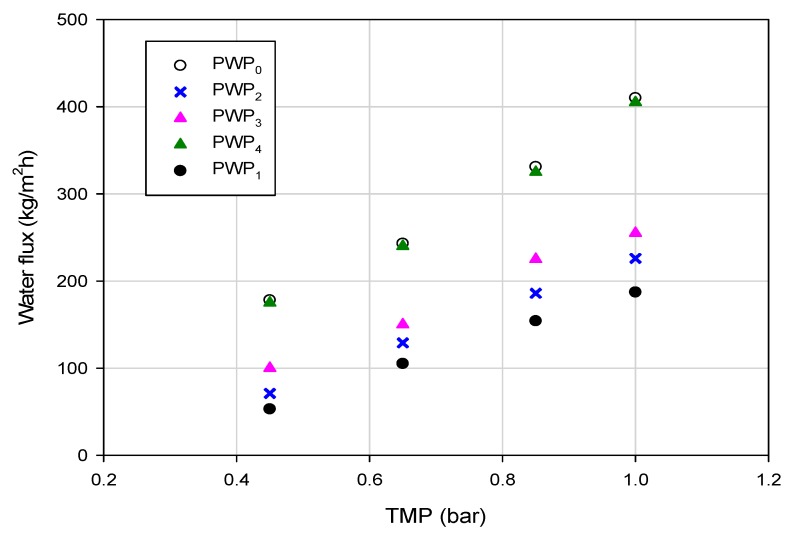
Pure water permeability of PVDF HF membranes before and after washing cycles (PWP_0_, initial pure water permeability; PWP_1_, pure water permeability after treatment of aqueous extract; PWP_2_, pure water permeability after cleaning with distilled water; PWP_3_, pure water permeability after cleaning with sodium hypochlorite; PWP_4_, pure water permeability after enzymatic cleaning).

The initial water permeability (PWP_0_), of about 396 kg/m^2^hbar, was reduced to 174 kg/m^2^hbar after the treatment of the aqueous extract. The fouling index calculated according to Equation (3) was of 56.1%. The first cleaning with distilled water allowed to recover about 53% of the initial water permeability (213 kg/m^2^hbar), due to the removal of the reversible polarized layer. The chemical cleaning improved the water permeability up to 252 kg/m^2^hbar. The initial water permeability was totally recovered through the enzymatic cleaning (Figure 6).

### 3.4. Fractionation of the Clarified Extract

**Figure 7:**
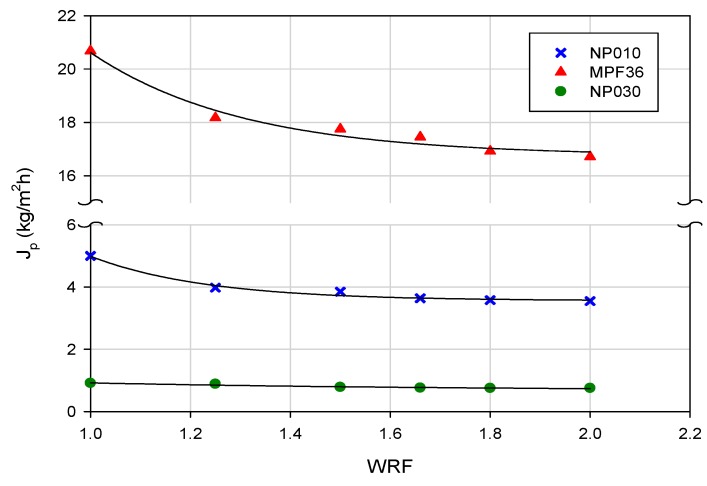
Treatment of clarified extract with NF flat-sheet membranes. Permeate flux as a function of WRF (TMP, 20 bar; T, 25 ± 2 °C).

The clarified extract was processed with three different membranes. The evolution of permeate flux as a function of WRF at an operating pressure of 20 bar for the selected membranes is reported in Figure 7.

Among the selected membranes the MPF36 showed the highest productivity with a steady-state permeate flux of about 17 kg/m^2^h. In particular, the membrane productivity decreased according to the following order:

MPF36 > NP010 > NP030

in agreement with the measured water permeability values (see Table 2).

For all membranes, the flux decay, and consequently, membrane fouling, was significantly minimized due to the preliminary clarification step which allowed to remove suspended solids improving the filterability of the aqueous extract. In particular, for the MPF36 membrane, the initial permeate flux of about 20.7 kg/m^2^h decreased by about 19% when a WRF 2 was reached. For the NP010 and NP030 membranes flux decays were of the order of 29% and 17%, respectively.

The NP030 membrane showed lower productivity when compared with the NP010 membrane (a membrane of similar composition but with higher MWCO), according to its lower MWCO. The MPF36 membrane showed a much higher productivity when compared with the NP010 membrane despite their similar MWCO. This behavior can be attributed to the higher hydrophilicity of the MPF36 membrane (contact angle 54°) in comparison to that of the NP010 one (contact angle 72°).

### 3.5. Analyses of Membrane Selectivity: Microfiltration of Red Wine Lees Extract

The physicochemical composition of permeate and retentate streams obtained in the clarification of the aqueous extract is reported in Table 4. The produced membranes removed all suspended solids retaining about 28.5% of soluble solids.

Most of the bioactive compounds, including phenolics, anhocyanins and resveratrol were recovered in the clarified extract. Indeed, despite the high fouling index of the MF membrane (56.1%), the retention of phenolic compounds, anthocyanins and resveratrol were of 15.3%, 11.2%, and 14.2%, respectively. This can be attributed to the large pore diameter of the MF membrane allowing the diffusion of compounds with low molecular weights [37]. Accordingly, the total antioxidant activity of the clarified solution was well preserved in comparison with the feed solution (the retention towards TAA was of 15.7%)

Low retentions towards phenolic compounds (3.1%) were also measured in the clarification of pomegranate juice by using PVDF HFs with a pore size of 0.13 μm [38]. PVDF membranes consist of alternating units of CH_2_ and CF_2_ conferring a hydrophobic nature to the material. Therefore, they are less prone to form hydrogen bonds and Van der Waals interactions with the hydroxyl groups exhibited by polyphenols and, therefore, more resistant to fouling and highly permeable to phenolic compounds when compared with polysulphone membranes.

### 3.6. Analyses of Membrane Selectivity: Nanofiltration of Clarified Extract

The physico-chemical characterization of permeate samples produced in the treatment of the clarified extract with NF membranes is reported in Table 5.

**Figure 8:**
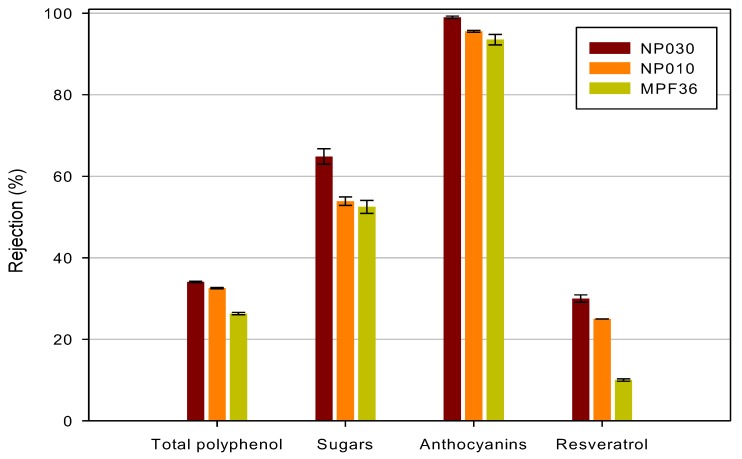
Rejection of NF membranes towards bioactive compounds and sugars.

The rejection coefficients measured towards different compounds for the investigated membranes are reported in Figure 8.

As a general trend, the rejection towards all detected compounds increased according to the following order:

MPF36 > NP010 > NP030

Anthocyanins retentions were significantly higher than 93% for all selected membranes with the NP030 exhibiting the highest value (99%): Therefore, these membranes appear to be suitable for concentrating the clarified extract and producing an added value concentrate of anthocyanins. Similar results were found by Cissé et al. [39] in the concentration of roselle extract (Hibiscus sabdariffa L.) with NF membranes, including those used in this work.

On the other hand, the retention of selected membranes towards phenolic compounds and resveratrol resulted lower than 34% and 30%, respectively. Accordingly, most part of phenolic compounds and resveratrol can be recovered in the permeate fraction. In particular, the MPF36 membrane exhibited the lowest retention towards resveratrol (10%) and polyphenols (26.3%) when compared with the other two membranes and the best separation factor between these compounds and anthocyanins.

Retention values for TAA were in the range of 29–35%, in agreement with those observed for polyphenols: These results corroborated the strict correlation between the phenolic content and the antioxidant activity measured in white and red wines [40].

The rejection of phenolic compounds can be explained not only on the basis of the MWCO of the selected membranes. Indeed, these compounds generally include aromatic (benzene) ring structures that have aliphatic carbon groups which, if undissociated in pH conditions lower than their pKa values, can be readily adsorbed on the membrane surface [41]. The increased hydrophobicity caused by the solute binding is accompanied by a decline of permeate flux through the membrane. In addition, an increase in membrane hydrophobicity (such as for PES membranes) leads to an increased adsorption of phenolic compounds [42]. The rule of plant phenols as adhesive coatings with long term stability has been also reported in the literature [43].

According to rejection data, the investigated membranes did not show a preferential rejection of phenolic compounds over sugars. Similar results were found by Diaz-Reinoso et al. [44] in the use of UF and NF membranes in the treatment of aqueous extracts from distilled fermented grape pomace.

The whole results indicate that the combination of MF and NF membranes is an attractive alternative process for producing, at low temperatures, concentrated extracts of anthocyanins from wine lees without thermal damage before final concentration (vacuum evaporation, osmotic evaporation) or spray drying. They can be considered a new source of anthocyanins for various applications in the food industry, as a natural colorant in alternative to synthetic ones, as nutraceuticals or in the pharmaceutical industry for new functional formulations.

Permeate streams containing resveratrol, and phenolic compounds appear also of interest for the formulation of natural products containing antioxidant compounds.

## 4. Conclusions

A green integrated approach applying sustainable extraction of red wine lees and membrane-based clarification and fractionation of aqueous extracts was proposed. PVDF membranes in hollow fiber configuration prepared according to the phase inversion method were characterized and used to clarify the aqueous extract. The prepared membranes showed an average pore diameter of 0.13 μm and a high porosity (81.0 ± 4.5 %) in agreement with their finger-like morphology. Steady-state permeate fluxes of about 53 kg/m^2^h were measured in the in optimized conditions of transmembrane pressure when the extract was processed according to a batch concentration configuration. Suspended solids were completely removed from the extract, producing a clarified solution enriched in sugars and bioactive compounds including phenolic compounds, anthocyanins, and resveratrol.

The clarified solution was then fractionated by using commercial NF membranes in flat-sheet configuration. All selected membranes resulted suitable for concentrating anthocyanins from the clarified extract (retention higher than 93%) with the NP030 exhibiting the highest retention (99%). Therefore, these membranes appeared suitable to concentrate anthocyanins from clarified wine lees extracts at low temperature before final concentration by vacuum evaporation or spray drying. Final products could be of interest for the use as natural colorants and/or for nutraceutical applications. Among the selected membranes, the MPF36 showed the lowest retention towards phenolic compounds and resveratrol (26.3% and 10%, respectively) with a production of a permeate fraction of interest for the formulation of natural antioxidant-based products.

The innovative integrated approach allows to obtain individual fractions enriched in bioactive compounds of interest for specific applications in pharmaceutical, cosmetic, food, and functional food industries. Overall, it turns up as a clean and a low-cost method to valorize red wine lees through the recovery of natural ingredients within the logic of both process intensification and zero discharge strategies.

## Figures and Tables

**Table 1:** Spinning conditions of PVDF HF membranes.

**Dope composition (wt.%)**	PVDF/DMF/PVP K17 25/40/35
**Dope flow rate (g/min)**	9
**Dope temperature (°C)**	120
**Bore fluid composition (wt.%)**	DMF/H_2_O 30/70
**Spinneret dimensions (mm)**	O.D./I.D. 1.6/0.6
**Bore fluid temperature (°C)**	50
**Outer coagulant**	Tap water at room temperature
**Air gap (cm)**	25

**Table 2:** Characteristics of selected membranes according to manufacturers unless otherwise stated.

Membrane Type	NP030	NP010	MPF-36
Manufacturer	Microdyn Nadir	Microdyn Nadir	Koch Membrane Systems
Membrane material	Polyethersulphone	Polyethersulphone	Polyamide
Configuration	Flat-sheet	Flat-sheet	Flat-sheet
Nominal MWCO (Da)	300–400	1000	1000
pH operating range	0–14	0–14	1–13
Operating temperature (°C)	5–95	5–95	5–70
Max. operating pressure (bar)	35	20	25
Contact angle (°)	88.4 ^a^	72 ^a^	54 ^b^
Water permeability at 25 °C (kg/m^2^hbar) ^c^	0.73	3.0	7.19

^a^ data from Boussou et al. [23], ^b^ data from Mänttäri et al. [24], ^c^ own measurements.

**Table 3:** Properties of PVDF HF membranes.

**Dimensions**	Outer diameter (mm)	2.29 ± 0.54
	Inner diameter (mm)	1.30 ± 0.65
	Thickness (mm)	0.49 ± 0.9
**Mechanical tests**	Emod (N/mm^2^)	32.47 ± 2.91
	εbreak (%)	104.33 ± 3.01
**Porosity (%)**		81.0 ± 4.5
**Pore size**	Bubble point (bar)	1.2
	Average pore diameter (μm)	0.13

**Table 4:** Physicochemical properties of feed, permeate and retentate samples obtained in the clarification of wine lees extract with hollow fiber membranes.

Parameter	Feed	Permeate	Retentate	Retention (%)
TSS (°Brix)	3.5 ± 0.1	2.5 ± 0.1	4.5 ± 0.4	28.5
Total suspended solids (%)	6.4 ± 0.2	n.d.	19.3 ± 1.4	100
TAA (mM Trolox)	3.8 ± 0.9	3.2 ± 0.1	2.9 ± 0.6	15.7
Total polyphenols (mg/L gallic acid)	1160.7 ± 5.6	982.1 ± 1.7	1331.6 ± 3.6	15.3
Sugars (mg/L glucose)	23.8 ± 0.3	16.8 ± 1.0	28.6 ± 0.1	29.8
Anthocyanins (mg/L cyanidin 3-glucoside)	369.1 ± 17.8	328.9 ± 6.1	434.1 ± 0.7	11.2
Resveratrol (mg/L)	0.29 ± 0.06	0.24 ± 0.02	0.30 ± 0.04	14.2

**Table 5:** Physicochemical properties of permeate samples obtained in the treatment of clarified extract with NF membranes.

Parameter	Feed	Permeate		
		NP030	NP010	MPF36
TSS (°Brix)	2.1 ± 0.02	0.8 ± 0.02	1.0 ± 0.04	1.3 ± 0.05
TAA (mM Trolox)	2.1 ± 0.3	1.4 ± 0.2	1.5 ± 0.2	1.5 ± 0.2
Total polyphenols (mg/L gallic acid)	994.9 ± 10.2	655.6 ± 5.1	670.1 ± 6.5	732.1 ± 6.1
Sugars (mg/L glucose)	12.96 ± 0.38	4.56 ± 0.38	5.98 ± 0.31	6.16 ± 0.39
Anthocyanins (mg/L cyanidin 3-glucoside)	322.12 ± 6.66	3.15 ± 1.02	14.35 ± 1.02	20.92 ± 4.59
Resveratrol (mg/L)	0.20 ± 0.004	0.14 ± 0.001	0.15 ± 0.003	0.18 ± 0.003
